# Conventional cardiovascular risk factors associated with Takotsubo cardiomyopathy: A comprehensive review

**DOI:** 10.1002/clc.23661

**Published:** 2021-06-03

**Authors:** Jing Liang, Jingyi Zhang, Yidan Xu, Catherine Teng, Xiaojia Lu, Yanxuan Wang, Xinyu Zuo, Qiuyue Li, Zirui Huang, Jianjun Ma, Pengyang Li

**Affiliations:** ^1^ Human Anatomy Laboratory, School of Basic Medicine Xinxiang Medical University Henan China; ^2^ Human Anatomy Laboratory, the First Clinical College Xinxiang Medical University Henan China; ^3^ Department of Medicine Yale New Haven Health‐Greenwich Hospital Greenwich Connecticut USA; ^4^ Department of Cardiology The First Affiliated Hospital of Shantou University Medical College Guangdong China; ^5^ Human Anatomy Laboratory, School of International Education Xinxiang Medical University Henan China; ^6^ Department of Medicine Saint Vincent Hospital Worcester Massachusetts USA

**Keywords:** age, cardiovascular risk factors, chronic kidney disease, diabetes, gender, hyperlipidemia, hypertension, obesity, Takotsubo cardiomyopathy

## Abstract

Takotsubo cardiomyopathy (TCM), characterized by transient left ventricular dysfunction, was first reported in Japan in 1990. Current research suggests that TCM can be affected by conventional cardiovascular factors such as hypertension (HTN), diabetes, hyperlipidemia (HLD), and obesity. Despite the increasing interest in this disease, research on TCM remains limited. Conventional cardiovascular factors are clinically related to the outcome of TCM. We reviewed the publications published in PubMed database between January 01 2010 and January 15 2021, and summarized the most current available evidence on the correlation between TCM and the conventional cardiovascular factors. TCM patients are predominantly postmenopausal women. Men and young patients are less commonly seen, but are prone to acute adverse complications and poor prognosis. HTN is common in patients with recurrent TCM. Existing evidence suggests that obesity and chronic kidney disease are related to poor prognosis in TCM. HLD is reported to be associated with fewer complications, though current evidence is limited. Finally, the relationship between diabetes and TCM prognosis is ambivalent. Current evidence suggests conventional cardiovascular risk factors are associated with the outcome of TCM, especially with mortality and complications. More prospective studies are needed to clarify the relationship between each risk factor and the prognosis of TCM.

**Abbreviations**
ACS: acute coronary syndrome
AKI: acute kidney injury
CI: confidence interval
CKD: chronic kidney disease
DKA: diabetic ketoacidosis
DM: diabetes mellitus
eGFR: esti glomerular filtration rate
ESC: European society of cardiology
HLD: hyperlipidemia
HR: hazard ratio
HTN: hypertension
Inter TAK: International Takotsubo
MeSH: Medical Subject Headings
OR: odds ratio
P: p value
SO: sympathetic overactivity
STEMI: ST‐segment elevation myocardial infarction
TCM: Takotsubo Cardiomyopathy

## 1 INTRODUCTION

Takotsubo cardiomyopathy (TCM) is a reversible left ventricular dysfunction that is commonly known as “broken heart” syndrome. The first case of TCM was in Japan in 1990, and has since become increasingly reported worldwide.^1^ TCM is a syndrome characterized by transient left ventricular systolic dysfunction that is similar to acute coronary syndrome (ACS), but without angiographic evidence of obstructive coronary artery disease or acute plaque rupture.^2^, ^3^ TCM was initially considered as a benign disease. However, recent research has found that the long‐term mortality rate of TCM patients exceeds that of ST‐segment elevation myocardial infarction (STEMI) patients.^4^ The pathogenesis of TCM is still unclear, which may involve a multi‐factor pathophysiological mechanism. At present, the most accepted hypothesis is the catecholamine surge, which lead to myocardial injury. Other proposed pathogenesis includes sympathetic overactivation (SO), coronary artery vasospasm, microvascular dysfunction, estrogen deficiency, and endothelial dysfunction.^5^, ^6^, ^7^, ^8^ There are different criteria for the diagnosis of TCM, and the Mayo Clinic diagnostic standard modified in 2008 is the most widely known.^9^ The new assessment method of the International Takotsubo (Inter TAK) diagnostic score has also been proposed recently.^10^

Studies demonstrated that TCM has distinct features in gender and age distribution, and its patient population is prominently postmenopausal women. In addition, conventional cardiovascular factors are relatively common in TCM patients, such as hypertension (HTN), hyperlipidemia (HLD), obesity, diabetes mellitus (DM), and chronic kidney disease (CKD).^11^, ^12^ However, evidence on the relationship of traditional cardiovascular factors and TCM are still limited. In this review article, we sought to summarize the current literature on the clinical relationship between TCM and conventional cardiovascular risk factors.

## 2 METHOD

We searched publications in the PubMed database published between January 01 2010 and January 15 2021. The following search terms were used, including Medical Subject Headings (MeSH): Takotsubo cardiomyopathy, Takotsubo syndrome, broken heart syndrome, stress cardiomyopathy, cardiovascular risk factors, hypertension, high blood pressure, diabetes mellitus, hyperlipidemia, obesity, CKD, age, gender and sex. Two investigators (J. L. and Y. X.) independently performed the literature research. Large prospective and retrospective studies that reporting mortality rate were included, and select case reports were also considered supplement the limited evidence available within the research topic.

## 3 RESULTS

**FIGURE 1:**
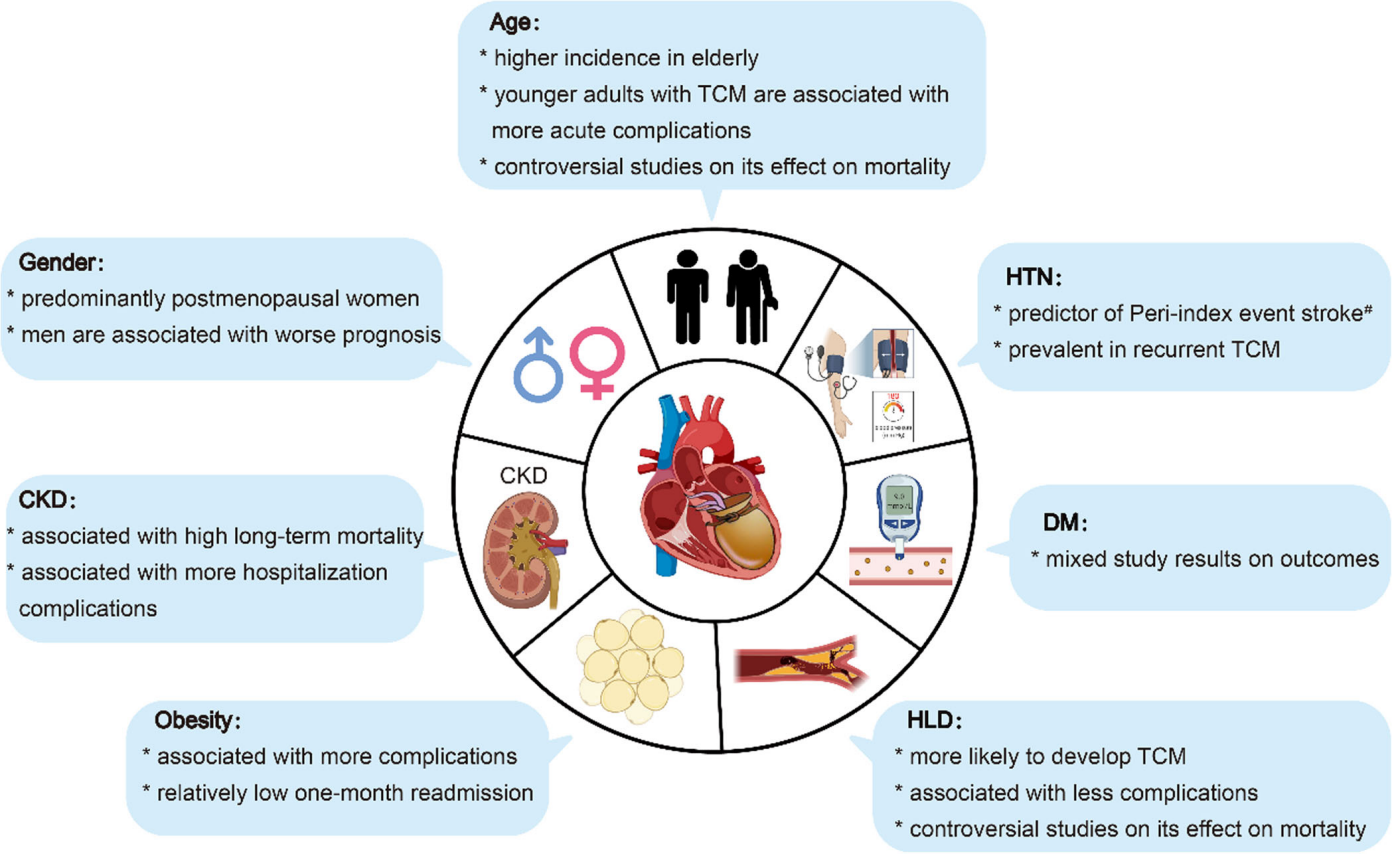
Conventional cardiovascular risk factors associated with Takotsubo cardiomyopathy. CKD, chronic kidney disease; DM, diabetes mellitus diabetes; HTN, hypertension; HLD, hyperlipidemia. ^#^New focal neurological symptom occurring during the hospitalization or up to 30 days after discharge

Selective risk factors, including HTN, DM, HLD, obesity, CKD, gender, and age, and the current evidence of their association with TCM outcome are discussed below in detail and summarized in Table 1 and Figure 1.

**TABLE 1:** The association of cardiovascular risk factors and TCM outcomes

Risk factor	Study type	No. of patients	Inclusion criteria (TCM)	Follow‐up	Outcome	Result	Reference
DM	DM vs. non‐DM						
	Prospective study	826	Modified Mayo Criteria and ESC Criteria (2016)	28‐day	Mortality CS Pulmonary edema	No difference, p = .772 No difference, p = .330 7.5% vs.3.7%, p = .032	Stiermaier T et al. (2018)^21^
				2.5 years (median)	Mortality	31.4%vs.16.5%, p < .001	
	Retrospective study	154	Modified Mayo Criteria	In‐hospital	Composite endpoints^a^	OR:2.92, 95%CI:1.01–8.41, p = .04	Kato Ken et al. (2018)^55^
	Retrospective study	206	Modified Mayo Criteria and ESC Criteria (2016)	In‐hospital	Mortality AF (new onset) HF	4.9% vs.7.9%, (p was not reported) 2.4% vs. 9.7% 24% vs. 27%	Dias A et al. (2016)^30^
Obesity	Obesity vs. non‐obesity						
	Retrospective study	1140	ICD‐9‐CM	In‐hospital	Mortality AMI CA CS CHF RF VTE Arrhythmia	No difference, P = .35 9.0% vs. 7.4%, p = .04 2.3% vs. 0.4%, p < .001 4.3% vs. 3.2%, p = .03 5.0% vs. 3.8%, p = .02 12.9% vs. 11.0%, p = .021 No difference, p = .952 No difference, p = .123	Desai R et al. (2018)^33^
	Retrospective study	5997	ICD‐9‐CM	1 months	readmission	OR:0.71, 95%CI:0.52–0.96, p = .027	Shah M et al. (2018)^34^
CKD	CKD^b^ vs. non‐CKD						
	Retrospective study	24 595	ICD‐9‐CM	In‐hospital	Mortality AKI requiring dialysis AKI CS, CA, VT and VF, ARF, ischemic stroke, post‐operative deep vein, thrombosis, pulmonary embolism, post‐operative sepsis	No difference, p = .269 6.34% vs. 1.36%, p < .0001 44.96% vs. 22.73%, p < .0001 No difference, All p > .05	Yassin AS et al. (2019)^35^
	Retrospective study	95	Modified Mayo Criteria	In‐hospital	Mortality Pneumonia, RF, urinary tract infection, rhythm disturbances, cardiac rupture	No difference, p = .42 No difference, All p > .05	Zalewska‐Adamiec M et al. (2018)^36^
				1 ‐year	Mortality	No difference, p = .72	
				3 ‐year	Mortality	33.3% vs. 15.4%, p = .047	
Gender	Male vs. Female						
	Retrospective study	39 662	ICD‐9‐CM	In‐hospital	Mortality CS VF or VT AKI	3.7% vs. 1.1%, p <.001 6.9% vs. 4.0%, p = .03 6.2% vs. 3.5%, p = .02 9.9% vs. 5.9%, p < .001	Lemor A et al. (2018)^41^
	Retrospective study	7510	ICD‐9‐CM	In‐hospital	Mortality VA SCD	4.8% vs. 2.1%, p = .04 No difference, p = .27 5.6% vs. 1.9%, p < .01	Krishnamoorthy P et al. (2015)^42^
	Prospective study	1750	Modified Mayo Criteria	30‐day 9.2‐year	Mortality MACCE^c^ Mortality MACCE	12.2% vs. 5.2%, p = .001 13.7% vs. 6.3%, p = .002 12.9% vs. 5.0%, p < .001 16.0% vs. 8.7%, p = .002	Templin C et al. (2015)^43^
	Retrospective study	24 701	ICD‐9‐CM	In‐hospital	Mortality CS VF/CA Acute CHF	8.4% vs. 3.6%, p < .0001 5.7% vs. 4.6%, p < .05 4.1% vs. 2.6%, p < .001 26.3% vs.31.1%, p <.001	Brinjikji W et al. (2012)^54^
	Retrospective study	102	unclear	In‐hospital	Mortality In‐hospital onset	No difference 77% vs. 17%, p < .01	Kurisu S et al. (2010)^45^
Age	≤50 vs. 51–74 vs. ≥75 years						
	Retrospective study	40 326	ICD‐9‐CM	In‐hospital	Mortality CS CA VA	1.1% vs. 1.0% vs. 1.9%, p < .001 11.9% vs. 4.8% vs. 3.4%, p < .001 3.3% vs. 1.1% vs. 0.8%, p < .001 6.8% vs. 3.2% vs. 3.5%, p < .001	Nazir S et al. (2020)^53^
	≤50 vs. 51–74 vs. ≥75 years						
	Prospective study	2098	Modified Mayo Criteria	In‐hospital	Mortality Acute neurological Psychiatric disorders CS	6.6% vs. 3.6% vs. 5.1%, p = .07 16.3% vs. 8.4% vs. 8.8%, p = .001 14.1% vs. 10.3% vs. 5.6%, p < .001 15.3% vs. 9.1% vs. 8.1%, p = .004	Cammann VL et al. (2020)^56^
	≤65 vs. > 65 years						
	Retrospective study	114	Modified Mayo Criteria	In‐hospital	Mortality Malignant arrhythmias Thromboembolic events	No difference, p = .73 No difference, p = .36 No difference, p = .77	Huseynov A et al. (2017)^52^
				30 days	Mortality	No difference, p = 1.00	
				4.4 ± 3.0 years (mean)	Mortality Recurrence	No difference, p = .70 No difference, p = 1.0	
	<65 vs. 65–74 vs. ≥75 years						
	Partially retrospective, partially prospective observational study	190	Modified Mayo Criteria	In‐hospital	Mortality Composite adverse events^d^	No difference, p = .24 17.1% vs. 22.2% vs. 37.5%, p = .03	Citro R et al. (2012)^57^
	<50 vs. 50–64 vs. > 64 years						
	Retrospective study	24 701	ICD‐9‐CM	In‐hospital	Mortality	No difference (p was not reported)	Brinjikji W et al. (2012)^54^

Abbreviations: AF, atrial fibrillation; AHF, acute heart failure; AKI, acute kidney injury; AMI, acute myocardial infarction; ARF, acute respiratory failure; CA, cardiac arrest; CHF, congestive heart failure; CI, confidence interval; CKD, chronic kidney disease; CS, cardiogenic shock; DM, diabetes mellitus; ESC, European Society of Cardiology; HF, heart failure; ICD‐9‐CM, International Classification of Diseases, 9th Revision, Clinical Modification; MACCE,major adverse cardiac and cerebrovascular events; OR, odds ratio; RF, respiratory failure; SCD, sudden cardiac death; TCM, Takotsubo cardiomyopathy; VA, ventricular arrhythmia; VF, ventricular fibrillation; VT, ventricular tachycardia; VTE, venous thromboembolism.

^a^ Including pulmonary edema, CS, sustained ventricular tachycardia or VF, complete atrioventricular block, thromboembolism, cardiac rupture, and cardiac death.

^b^ CKD is defined as eGFR<60 mL/min/1.73 m^2^.

^c^ A composite of a recurrence of takotsubo cardiomyopathy, myocardial infarction, stroke or transient ischemic attack, or death from any cause.

^d^ Including all‐cause death, AHF, life‐threatening arrhythmias, stroke, and CS.

### 3.1 Hypertension

HTN is common in TCM. In a systematic review of 1109 TCM patients, 54% of them carry a diagnosis of HTN.^11^ Research on the association between HTN and TCM is limited to retrospective studies and the result is mixed. In a study of 6837 TCM patients, HTN was found to have had no association with the incidence of TCM.^13^ On the other hand, existing HTN can predict a worse prognosis of TCM, as demonstrated by a retrospective descriptive study of 206 TCM patients from 2003 to 2014, which found that HTN was an independent predictor of peri‐index event stroke (new focal neurological symptom occurring during the hospitalization or up to 30 days after discharge) in TCM patients (OR:10.5, 95%CI:1.3–88, *p* = .03).^14^ The case of acute hypertensive crisis induce TCM has also been reported.^15^

HTN is more prevalent in recurrent TCM patients compared to non‐recurrent cases. A prospective study of 749 TCM patients showed that the incidence of HTN in the TCM recurrence group was significantly higher than those in the non‐recurrence group (86.7% vs. 68.3%, p = .03).^16^ Similar finding was noticed in another retrospective study of 114 TCM patients (100% vs. 55.1%, p = .02).^17^

HTN, as a typical manifestation of pheochromocytoma,^18^ can be associated with the potential trigger of TCM in the setting of catecholamine surge.^19^ In a review of 38 TCM patients secondary to pheochromocytoma, the incidence of HTN in pheochromocytoma‐induced TCM group was 52.6%.^20^

### 3.2 Diabetes mellitus

DM is common in TCM patients, with its prevalence ranging from 12.6% to 22.8%.^21^, ^22^, ^23^, ^24^

Though cases have been reported with complications of DM triggering TCM, the clear association between the two diseases are not well established. Diabetic ketoacidosis (DKA),^25^ DKA induced hypothermia,^26^ and DKA with thyroid storm^27^ have been identified as causes of TCM in case reports so far.

Research on DM's effect on TCM outcome has generated conflicting results (Table 1). A 3.8 years follow‐up study showed that DM was an predictor of long‐term mortality (HR:2.11, 95%CI:1.23–3.65, p < .01).^4^ Another retrospective study showed that DM was an predictor of 90‐day TCM readmission (OR:1.36, 95%CI:1.27–1.47, p < .001).^28^ In a prospective study of 826 TCM patients, the mortality was significantly higher in DM‐TCM group than non‐DM‐TCM group (31.4% vs. 16.5%, p < .001) after on average 2.5 years follow up.^21^ In the same study, DM was also an independent predictor of adverse outcomes (HR:1.66, 95%CI:1.16–2.39, p = .006).^21^ Similar to prior findings, a study of 178 TCM patients without cardiogenic shock, DM was found to be an independent predictor of overall mortality after 3.6 years follow‐up (HR:2.05, 95%CI:1.07–3.94, p = .03).^29^

Counterintuitively, some retrospective studies argued that DM had a positive association with TCM outcome. A single‐center retrospective observational study of 114 TCM patients reported that the rate of cumulative event within 1 year, including all‐cause death, life‐threatening arrhythmias, thromboembolic events, rehospitalization for heart failure, TCM recurrence rate and stroke, were significantly lower in DM‐TCM group compared to non‐DM‐TCM group (p = .04).^23^ In a retrospective descriptive study of 206 TCM patients, the DM‐TCM group (n = 41) had a lower incidence of inpatient heart failure (24% vs. 27%), and a lower inpatient mortality rate (4.9% vs. 7.9%), though the big drawback of the study was the unreported of p‐value of its statistical analysis.^30^

### 3.3 Hyperlipidemia

The prevalence of HLD in TCM is thought to be as high as 43%.^14^ Our literature review showed limited data on the association between HLD and TCM occurrence, and HLD and TCM outcomes. A retrospective study of 6837 TCM patients found that HLD was associated with the higher odds of developing TCM after adjusting for comorbidity (p < .05).^13^ The impact of HLD on the prognosis of TCM patients is yet to be determined. In a retrospective cohort study, HLD‐TCM group had lower rates of in‐hospital mortality (1.1% vs. 2.4%, p = .027), acute respiratory failure (9.1% vs. 12.1%, p = .022) and cardiogenic shock (3.4% vs. 5.6%, p = .012), shorter length of stay (3.20 ± 3.27 vs. 3.57 ± 3.14 days, p = .005), and lower total charges (p = .013) compared to non‐HLD‐TCM group.^31^ However, another study using the same database, found HLD was not associated with the in‐hospital mortality in TCM patients though the mortality rate in each group was not reported (p = .147).^32^ The current research on HLD and TCM is still minimal, further research is needed.

### 3.4 Obesity

Nearly 9.7% of TCM patients are obese, defined as body mass index over 30 kg/m^2^.^33^ The available studies on the prognostic impact of obesity on TCM patients are limited and summarized as below. (Table 1).

In a retrospective cohort study of 1140 TCM patients, obese TCM patients were more likely to have major complications, such as acute myocardial infarction (9.0% vs. 7.4%, p = .04), cardiac arrest (2.3% vs. 0.4%, p < .001), and cardiogenic shock (4.3% vs. 3.2%, p = .03), although there was no significant difference in all‐cause hospital mortality, length of stay, and hospitalization expenses (p > .05).^33^ Counterintuitively, another retrospective study of 5997 TCM patients revealed that obesity predict a lower 1‐month readmission rate (OR:0.71, 95%CI:0.52–0.96).^34^

### 3.5 Chronic kidney disease

CKD is also common in TCM patients, with 6.7% TCM patients carrying this diagnosis, according to a large retrospective study of 24 595 patients.^35^ Among CKD‐TCM patients, the creatine kinase (806.6 ± 1590 vs. 269.8 ± 312.7 IU/L, p < .001), fibrinogen (4.81 ± 2.4 vs. 3.803 ± 1.02 g/L, p = .03) and C‐reactive protein level (55.8 ± 78.6 vs. 21.6 ± 23.7 mg/L, p = .03) are significantly higher.^36^ Atrial fibrillation (13.3% vs. 1.54%, p = .02) and prolonged QTc (486.4 ± 38.8 vs. 462.8 ± 37.3 ms, p = .007) were also observed more frequently in CKD‐TCM patients.^36^

Current retrospective studies suggest that pre‐existing CKD is not associated with in‐patient mortality difference in TCM patients, though the long‐term mortality might be higher in CKD‐TCM patients compared to TCM patients without CKD. A retrospective study of 24 595 TCM patients found no statistical significance in the inpatient mortality between TCM patients with advanced CKD (eGFR<60 ml/min/1.73 m^2^) and those without advanced CKD (eGFR>60 ml/min/1.73 m^2^) (p = .269).^35^ Another retrospective study of 95 TCM patients further illustrated the lack of in hospital mortality (p = .42) and 1‐year mortality difference (p = .72) in CKD‐TCM group compared to non‐CKD‐TCM group. The 3 years mortality was significantly higher in CKD‐TCM group (33.3% vs. 15.4%, p = .047).^36^

TCM patients with CKD are more likely to have inpatient complications (Table 1). In a retrospective study of 61 TCM patients, CKD‐TCM patients were more likely to have inpatient complications, including all‐cause mortality and severe pump failure, than non‐CKD‐TCM patients (HR:2.49, 95%CI:1.01–5.98).^37^ A multicenter observational study also showed that CKD was an independent predictor of hospital complications, such as acute heart failure and atrial fibrillation, in TCM (OR:7.99, 95%CI:1.39–45.79, p = .02).^38^ Another retrospective study of 24 595 TCM patients suggested that TCM patients with advanced CKD had a higher risk of acute kidney injury (AKI) (44.96% vs. 22.73%, p < .0001). The length of hospital stay was also more prolonged in advanced CKD patients (OR:1.12, 95%CI:1.03–1.22, p = .01),^35^ which is again demonstrated in another retrospective study of 219 TCM patients (20 ± 25 vs. 13 ± 15 days, p = .024).^39^

### 3.6 Gender

TCM patients are predominantly postmenopausal women.^16^, ^40^, ^41^, ^42^ The ratio of women to men in TCM patients is approximately 9:1 (89.8%–91.7% female).^13^, ^41^, ^43^ Rarely, in pediatric population, the study available did not show similar gender distribution (52.9% female).^44^

Women have a high tendency to develop TCM, but inpatient morbidity in male TCM patients is higher (77% vs. 17%, p < .01).^45^ Consistent research results point to a higher the inpatient mortality in males (Table 1). The rate of inpatient mortality in men was almost four times than that of women in a retrospective study (3.7% vs. 1.1%, p < .001).^41^ Similarly, another study of 82 TCM patients by Sobue Y et al. showed that being men was an independent predictors of hospital mortality in TCM patients (HR:11.9, 95%CI:2.43–58.5, p = .002).^46^ A larger study of 1750 TCM patients indicated that men had a higher risk than women of major adverse cardiovascular and cerebrovascular events within 30 days after admission (13.7% vs. 6.3%, p = .002).^43^ A similar finding was indicated in a study of 368 TCM patients which indicated that men were independent predictors of adverse composite cardiac events, including cardiovascular death, severe pump failure, and severe ventricular arrhythmia (OR:4.32, 95%CI:1.41–13.6, p = .011).^47^ One possible explanation of this phenomenon is that men with TCM are more commonly affected by physical stress.^45^ The study by Sobue Y et al. also found that the inpatient mortality rate of TCM patients with physical stress was higher than that of patients without physical triggers (20.9% vs. 2.6%, p = .007).^46^

### 3.7 Age

In TCM, men in general (50‐72 years) are younger than women (70–76 years).^47^, ^48^ TCM cases have been reported in children,^49^ and even in a premature neonate born in the 28th gestational week.^50^ In addition, women are significantly more likely to develop TCM after 55 years of age. This is demonstrated in a retrospective study by Deshmukh et al, where women older than 55 years were 4.8 times more likely to develop TCM than women younger than 55.^13^

Limited studies have conducted to look at age's influence on the TCM mortality, and the current evidence available is yet to generate a consensus on this matter (Table 1). A prospective study with on average 5.8‐year follow‐up showed that increased age was an independent predictor of TCM mortality (HR:1.059, 95%CI:1.037–1.081, p < .001).^51^ The sample size of the study was 265. Another retrospective study of 114 TCM patients showed no statistically significant association between the long‐term TCM mortality and age difference (p = .60). The mean follow up time was 1591 days.^52^

Younger patients are prone to have more complications, as compared to older age group. A retrospective study of 40 326 TCM patients showed the younger group was more likely to suffer from cardiogenic shock (11.9% vs. 4.8% vs. 3.4%, p < .001), cardiac arrest (3.3% vs. 1.1% vs. 0.8%, p < .001), and ventricular arrhythmia (6.8% vs. 3.2% vs. 3.5%, p < .001), compared with the middle‐aged group and the elderly group. The young, middle‐age, and elderly group were defined by age no more than 50 year‐old, age 51–74 year‐old, and age no less than 75 year‐old respectively. Young age was independently associated with risk of cardiac arrest (OR:2.92, 95% CI:2.33–3.63), and ventricular arrhythmias (OR:2.09, 95% CI:1.81–2.43).^53^

## 4 CONCLUSION

Primarily manifested as ACS, TCM shares the conventional cardiovascular risk factors, similar to the other major cardiovascular events. The risk factors include but are not limited to HTN, DM, HLD, obesity, CKD, gender, and age. The impact of conventional cardiovascular risk factors on the mortality and complications of TCM is largely undefined, owing to the limited large sample sized studies available. Through our literature review, we found that conventional cardiovascular factors as stated above were relatively common in TCM. There are obvious gender differences in TCM patients as the majority of patients are post‐menopausal women. Male patients and younger patients are less common, but they are more prone to acute adverse complications and have a poor prognosis. HTN is more prevalent in recurrent TCM patients. The existing research indicated that obesity and CKD are related with a poor prognosis of TCM. On contrary, HLD was noted to be associated with less complications in TCM patients, though current evidence is limited. Lastly, DM poses unclear associations with TCM prognosis. More high quality, large sample size studies are required to further clarify each risk factor's association with TCM short‐term and long‐term outcomes.

## CONFLICTS OF INTEREST

The authors report no conflicts of interest.

## Data Availability

The data supporting this review are from previously reported studies and datasets, which have been cited.
